# Work–family life course patterns and work participation in later life

**DOI:** 10.1007/s10433-018-0470-7

**Published:** 2018-04-02

**Authors:** Mai Stafford, Rebecca Lacey, Emily Murray, Ewan Carr, Maria Fleischmann, Stephen Stansfeld, Baowen Xue, Paola Zaninotto, Jenny Head, Diana Kuh, Anne McMunn

**Affiliations:** 1 MRC Unit for Lifelong Health and Ageing at UCL, 33 Bedford Place, London, WC1B 5JU UK; 2 Department of Epidemiology and Public Health, UCL, London, UK; 3 Wolfson Institute of Preventive Medicine, Queen Mary University of London, London, UK

**Keywords:** Multichannel sequence analysis, Longitudinal, Extending working lives, Retirement

## Abstract

Many developed nations seek to increase older people’s work participation. Work and family are linked to paid work in later life, and to each other. Few studies combined work and family histories using multichannel sequence analysis capturing status and timing of transitions in relation to work in later life. Using the MRC National Survey of Health and Development, for whom State Pension Age was age 65 (men) or 60 (women), we examined paid work at age 60–64 (and age 68–69 for men only) by work–family patterns across 35 years (ages 16–51). Women’s later work was related to the combination of timing of children and work during family formation. Women who had children later were more likely to work full-time at age 60–64 compared to the reference [characterised by continuous full-time employment, marriage, and children from their early 20s; adjusted OR 5.36 (95% CI 1.84, 15.60)]. Earlier motherhood was associated with lower likelihood of work at age 60–64 among those who did not return to work before age 51, but those who took a work break did not differ from those who worked continuously. Providing jobs which allow parents to combine work and family (e.g. part-time jobs) may encourage them to extend their working lives. In addition, men and women characterised by continuous full-time work and no children were less likely to work in their sixties. Associations were not explained by childhood health and social class, education, caregiving, housing tenure, or limiting illness. Research is needed to understand why childless people work less in later life.

**Electronic supplementary material:**

The online version of this article (10.1007/s10433-018-0470-7) contains supplementary material, which is available to authorized users.

## Introduction

Governments in many developed nations seek to increase the paid work participation rates of older people. This is in response to the projected increase in the number of people aged 65 and over, and concerns over rising dependency ratios (Eurostat 2017). In Britain, initiatives to increase work participation among people in their 50s and 60s are a priority and the State Pension Age is being raised (Department for Work and Pensions 2014). Many post-war baby-boomers (born between 1945 and 1965) have now retired and did so before their State Pension Age. Subsequent generations will be required to extend their working life because of the increase in the State Pension Age. Evidence on the factors that facilitate early and late exit is important for researchers and policy to provide advice on effective ways to extend working lives.

Work and family experiences across the life course, such as hours of working, partnerships and childrearing, have been identified as key factors throughout adulthood that cumulatively stratify people according to socioeconomic resources and opportunities for work (Dannefer 2003; O’Rand 2009) and influence retirement decisions. Studies of employment histories indicate that paid work participation in earlier adulthood is positively associated with participation in the late career stage (Blekesaune et al. 2008; Finch 2014; Pienta 1999), supporting the theory of status maintenance across the life course (Pampel and Hardy 1994). Women who have more family-related work interruptions are less likely to be in paid work in later life (Hank and Korbmacher 2013; Finch 2014; Pienta et al. 1994; Pienta 1999). Women’s weaker behavioural attachment to the labour market in earlier adulthood partly explains gender differences in later life work participation (Worts et al. 2016).

Family histories are also related to later life employment. Having children has been associated with greater likelihood of working in later life compared with not having children (Blekesaune et al. 2008; Hank 2004; Pienta 1999). Explanations typically refer to the opportunity costs of taking time out of paid employment to raise a family. This may lead people to work into later life due to lower levels of accumulated pension wealth. This cost is primarily born by women, though the cost of financially supporting children falls upon fathers as well as mothers and this might explain the lower later life work participation of childless men. Financial responsibility for children might also explain why becoming a parent at an older age is positively associated with later life work participation (Blekesaune et al. 2008; Pienta et al. 1994; Pienta 1999). Among women, it is also proposed that having children, especially at a younger age, reflects greater family orientation and that it is orientation, rather than structural constraints following family formation, that is reflected in their later retirement decisions (Hakim 2002). The long-term consequences of number and timing of children for women’s retirement decisions might depend on their form of return to work following childbearing. Studies have previously found long-term differences in wages and subsequent employment in mothers according to the length of their parental leave and changes in their job or their working hours on return to work (Lalive and Zweimuller 2009; Phipps et al. 2001) though we are not aware that employment in later life has been an explicit focus to date.

The interlinking of work and family patterns has long been recognised in life course research (Elder 1985; Kruger and Levy 2001). Previous studies have accounted for the links between paid work and family factors in statistical models which mutually control for summary indicators of parenthood, partnership, and employment status and timing and their statistical interaction (Blekesaune et al. 2008; Moen and Smith 1986; Pienta 1999). However, summary indicators do not fully utilise longitudinal data on work and family status collected repeatedly across adulthood. Multichannel sequence analysis (Gauthier et al. 2010; Pollock 2007) is one analytical approach to model the combination of work and family status and timing of transitions across adulthood (Fasang 2012; Lacey et al. 2016; McMunn et al. 2015; Madero-Cabib and Fasang 2016; Madero-Cabib et al. 2016). Based on retrospective data from Swiss participants in SHARELIFE, this approach has been used to explore retirement timing according to patterns of employment, pension investments, marital status and parenthood status (Madero-Cabib et al. 2016). Whereas marriage and childbirth did not differentiate men’s employment patterns in that study, among women these were linked to work breaks and lower investments in pensions and ultimately greater likelihood of later retirement. In that study, timing of family formation did not emerge as a factor which differentiated the work–family types.

We build on previous studies by characterising people’s joint work–family patterns, including full-time and part-time work, whether or not they had children, and the timing of partnership, children and career breaks, from age 16 through to their early 50s using multichannel sequence analysis of data from a population-based British cohort study. We examine whether patterns are related to paid work in later life (age 60 +). In Britain, the State Pension Age was 60 years for women and 65 years for men in the cohort of interest (born in 1946). As noted, this is being raised for later cohorts (born in 1951 onwards). The majority of men and women born in the mid-1940s contributed to private (including occupational) as well as state pensions (Banks and Casanova 2003). Their average age of withdrawal from the labour market was 61.9 years for women and 64.4 years for men and this is also rising among later cohorts (Office for National Statistics 2013). Men and women are examined separately because women’s work–family patterns are more complex than men’s (McMunn et al. 2015) and gender may modify the impact of family on work. We control for selection into work–family patterns on the basis of childhood socioeconomic position, childhood physical or mental health, and education. Children who experience poor health or socioeconomic disadvantage are less likely to be in paid work in adulthood (Case et al. 2005). More highly educated men have historically been more likely to marry than their less well educated counterparts, but more highly educated women were less likely to marry (Kiernan and Eldridge 1987). We also control for other possible predictors of later life employment including later life illness, wealth, and caregiving.

We hypothesise that the likelihood of being in paid work aged 60 + will be (1) greatest for those whose adult lives are characterised by continuous employment and relatively late family formation; (2) lowest for those who spend relatively little of their adult lives in full-time work and who either formed their families earlier or did not partner or have children. We hypothesise that the difference in paid work age 60 + between those who do and do not have children will be greater for men than women. This is because both fathers and mothers may continue to work to financially support their children, but childrearing tends to reduce women’s paid work participation in earlier adulthood which may limit their job opportunities in later life.

## Methods

### Study population

The MRC National Survey of Health and Development (NSHD) is a social class stratified sample of 5362 births of all singleton births within marriage in a week in March 1946 in England, Scotland and Wales. The sample has been followed up approximately every 2 years in childhood and the main data collections in adult life were at 26, 36, 43, 53, 60–64 and 68–69 years. Ethical approval was obtained from the Greater Manchester Local Research Ethics Committee and the Scotland A Research Ethics Committee. Written, informed consent was obtained for each component of data collection.

In 2014–2015, study members completed a postal questionnaire at age 68 and had a home visit by a research nurse at age 69 (Kuh et al. 2016). Of the 2816 in the target sample living in Britain, information was obtained from 2638 (94%). In addition, a postal questionnaire was sent to 126 study members living abroad who remain in contact with the study of whom 86 (68%) returned a questionnaire. No attempt was made to contact the remaining 2420: 957 (18%) had died, 620 (12%) had previously withdrawn, 448 (8%) had emigrated and were no longer in contact, and 395 (7%) had been untraceable for more than 5 years.

### Participation in paid work

At ages 60–64 (actually 90.1% of participants were aged 62–64 at this visit) and 68–69, study members reported their employment status and usual working hours. At age 60–64, we categorised study members as being in full-time employment (> 30 h per week), part-time employment (1–30 h per week), or not in employment. At age 68–69, we categorised men as being in employment or not and did not differentiate part- and full-time working due to small numbers. We did not include women’s work participation at age 68–69 as only 11% were in paid work.

### Work–family patterns

Work, partnership, and parenthood data were collected in face-to-face interviews from age 16 to 51. Work was based on self-reported labour market status, categorised as ‘full-time employment’, ‘part-time employment’ (≤ 30 h/week), ‘homemaking’ (based on full-time caring for the home and family) or ‘other not employed’ (sick, in education, unemployed, retired, not working for any other reason). Dates of marriage, cohabitation (from age 36 onwards), separation/divorce and widowhood were categorised for each year as ‘married’, ‘cohabiting’, or ‘not living with a partner’. Parental status was based on having dependent children in the household and categorised as ‘no children under 17 years’, ‘youngest child aged 0–4 years’ or ‘youngest child aged 5–16 years’. Where work, partnership or parenthood status included more than one response in a given year, the modal activity based on months spent in that activity was used. These three domains were combined into a single work–family state (4 work states × 3 partnership states × 3 parenthood states = 36 possible combinations) at each age from 16 to 51 years. Work–family patterns were derived up to age 51 to ensure separation between our exposure and outcome of interest. Around 20% had retired from their main occupation by age 51 and mean age of retirement was 58 years.

Multichannel sequence analysis was used to group individuals into work–family types based on these three domains in combination. Each sequence is compared to all others in the dataset to measure the distinctness or similarity (Abbot and Tsay 2000), using information on states at each age, and calculating a set of distance measures representing the ‘cost’ (reflecting the number of substitutions and insertions or deletions needed) of converting one sequence to another (MacIndoe and Abbott 2004). Distances can be derived in two ways: (1) relative to every other sequence in the data using data-driven methods; or (2) relative to one or more reference sequences, often theoretically derived and aimed at grouping individuals on the basis of their closeness to a specified biography (Gauthier et al. 2010; Pollock 2007). We chose the latter approach to explicitly differentiate people according to whether or not they had children and the timing of their family events in combination with their work status and timing of work breaks. A priori, two of the authors independently designed a set of reference types based on existing research about key patterns of work and family (Ferri et al. 2003; McMunn et al. 2015; Lacey et al. 2016). The independently derived types were then compared and found to overlap substantially. Based on this overlap, a common set of reference types was agreed upon, resulting in eight patterns (Table 1; Supplementary Tables A, B). Each participant was allocated to the biography to which their observed work–family sequence was most similar based on the Dynamic Hamming algorithm, which accounts for timing of transitions (Lesnard 2010) using the SEQCOMP Stata plug-in (Lesnard 2008). Missing information on work–family histories was handled using multiple imputation whereby twenty imputed datasets were created using a method developed for categorical time series data (Halpin 2012, 2013). This uses observed data preceding and following the missing data point(s) including the length of time an individual has spent in preceding and subsequent states. For each gap, the initial missing data point incorporates preceding information and the terminal missing data point incorporates subsequent information. Maximum internal gaps of 18 years and maximum initial and terminal gaps of 9 years were imputed here.

**Table 1:** Combined work–family patterns and frequencies in the MRC National Survey of Health and Development based on annual paid work, parenthood, and partnership status between ages 16–51

Work–family pattern label	Description	Men (*n* = 1257) %	Women (*n* = 1256) %
Work, early family	Continuous full-time employment; marriage and children from early 20s	49.9	14.0
Work, marriage, non-parent	Continuous full-time employment; married from early 20s; no children	7.7	9.1
Work, no family	Continuous full-time employment; no partnership or children	9.8	5.8
Work, later family	Continuous full-time employment; cohabiting from mid 20s; married from late 20s; children from early 30s	30.3	3.4
Later family, work break	Employed full-time until late 20s, homemaking from early-mid 30s, employed full-time from mid-40s; married from mid-20s; children from early 30s	1.0	12.7
Early family, work break	Employed full-time until early 20s, homemaking from early-late 20s, employed part-time early-mid 30s, employed full-time from late 30s; children from early 20s	0.6	15.1
Early family, part-time work	Employed full-time until early 20s, employed part-time from mid 20s, marriage and children from early 20s	0.8	30.7
Early family, no paid work	Employed part-time until early 20s, homemaking from early 20s; marriage and children from early 20s	0.01	9.2

### Covariates

Childhood social class was based on father’s occupation at age 4 (or age 7 or 11 if this was missing) and coded according to the Registrar General’s Social Class schema [I/II ‘professional/managerial or technical’, IIINM ‘skilled non-manual’, IIIM ‘skilled manual’, and IV/V ‘partly skilled or unskilled manual’ occupations (OPCS 1991)]. Teacher ratings of adolescent behaviour at ages 13 and 15, based on a forerunner of the Rutter A scale with seven items, were used to derive a conduct problems score (Colman et al. 2009) and identify those with poor adolescent mental health (with a score in the top quintile at either age). Serious illness requiring hospitalisation for a period of 28 days or more before age 16 was reported by the mother. Educational attainment was captured by highest achieved qualification at age 26 and categorised as none, ordinary secondary (O-levels or equivalent typically attained at age 16), advanced secondary (A-level or equivalent typically attained at age 18), or higher qualifications (university degree level or equivalent). At ages 60–64 and 68–69, study members reported whether they provided any unpaid care for an ill or frail person (categorised as providing ≥ 10 h per week or not), their housing tenure (categorised as owning outright, buying their house with a mortgage, renting or other), and if they had any limiting long-term illness or disability.

### Statistical methods

Regression models were used to estimate associations between work–family patterns and employment status at age 60–64 (men and women separately) and 68–69 (men only). At age 60–64, employment status was modelled using multinomial regression with the response categories ‘Full-time’, ‘Part-time’ and ‘Not in paid employment’ (the base outcome). At age 68, employment status was modelled using logistic regression with response categories ‘In paid employment’ and ‘Not in paid employment’. Models were adjusted sequentially: (1) crude model, (2) adjusted for selection into work–family pattern based on childhood factors and education, (3) additionally adjusted for concurrent caregiving, housing tenure, and limiting illness. Due to small numbers of men in the four work–family patterns at the bottom of Table 1, they were not included in the regression models. In all models, the ‘work, early family’ pattern was taken as the reference category. This represents those with continuous full-time employment who partnered and had children from their early 20s and comprises almost 50% of men and 14% of women.

Covariates and outcomes were imputed in a second round of imputations using multiple imputation by chained equations. Employment outcomes were included in imputation models but not in the estimation models (Von Hippel 2007). The analytical sample is those with work–family histories at age 16–51 who have at least one observed outcome at age 60–64 or 68 (*n* = 2513). The analytical sample did not differ from others followed up to age 60 + on child or adult covariates. All analyses were conducted in Stata version 14 (StataCorp 2015).

## Results

Almost 50% of men were characterised by continuous full-time employment, marriage and children from their early 20s (Table 1). There was more variation in women’s biographies, the most prevalent being characterised by full-time employment until early 20s, children from early 20s, followed by homemaking then part-time employment, and full-time employment again from late 30s.

Over 35% of men and 65% of women were not in paid employment at age 60–64 (Table 2). Women in paid work at this age were more commonly in part-time jobs, whereas men were more commonly in full-time jobs. By age 68–69, only a minority of men (22%) and women (11%) were in paid employment.

**Table 2:** Characteristics of the analytical sample

	Both genders		Men	Women	Gender difference
	Observed (%)	Imputed (%)	Imputed (%)	Imputed (%)	*p* value^a^
*Employment outcomes*					
In paid work at age 60–64					
Working > 30 h/week	30.3	30.3	49.6	12.4	<0.001
Working ≤ 30 h/week	18.2	18.1	14.2	21.8	
Not working	51.5	51.6	36.2	65.8	
In paid work at age 68–69					
Yes	17.0	16.7	22.3	11.3	<0.001
No	83.0	83.3	77.7	88.7	
*Childhood covariates*					
Father’s social class					
I/II (most advantaged)	16.1	16.3	17.5	17.7	0.9
IIINM	10.5	10.8	11.1	11.8	
IIIM	44.4	44.0	44.2	43.2	
IV/V (least advantaged)	29.1	28.9	27.3	27.4	
Educational attainment					
No qualifications	42.4	42.1	38.0	39.8	<0.001
≤ O-level or equivalent	28.9	29.0	21.6	38.1	
A-level or equivalent	21.7	21.8	28.0	18.7	
Degree level	7.0	7.1	12.4	3.5	
Poor adolescent mental health					
No	79.9	80.2	84.0	78.1	<0.001
Yes	20.1	19.8	16.0	22.0	
Childhood illness requiring hospitalisation					
No	84.5	84.5	84.8	86.0	0.1
Yes	15.5	15.5	15.2	14.0	
*Concurrent covariates*					
Caregiving for ≥ 10 h/week at age 60–64					
No	91.2	90.8	92.4	89.2	<0.001
Yes	8.8	9.2	7.6	10.8	
Caregiving for ≥ 10 h/week at age 68–69					
No	92.0	91.6	93.3	90.6	<0.001
Yes	8.0	8.4	6.7	9.4	
Housing tenure at age 60–64					
Own outright	69.4	69.5	65.5	73.2	<0.001
Mortgage	20.6	20.6	25.0	16.5	
Rent/other	10.0	10.0	9.6	10.3	
Housing tenure at age 68–69					
Own outright	84.2	83.0	82.9	83.2	0.001
Mortgage	5.9	6.3	6.9	5.6	
Rent/other	9.8	10.8	10.2	11.2	
Long-term limiting illness at age 60–64					
No	74.7	74.2	75.3	74.1	<0.001
Yes	25.3	25.8	24.8	25.9	
Long-term limiting illness at age 68–69					
No	56.4	56.3	57.5	55.1	<0.001
Yes	43.6	43.7	42.5	44.9	

^a^*p* values calculated by Chi square test on imputed data

### Work and family formation

Men who neither partnered nor had children (‘work, no family’ pattern) were less likely to be in full-time work at age 60–64 (RRR = 0.37, 95% CI 0.21, 0.66), compared with the reference group of men in the ‘work, early family’ pattern (Table 3). This was not explained by childhood health, social class or education (Table 3, model 2) or by caregiving, housing tenure, and limiting illness at age 60–64 (Table 3, model 3).

**Table 3:** Likelihood of being in full-time or part-time employment at age 60–64 (base outcome is not in employment); men (*n* = 941)

	Model 1: crude association				Model 2: model 1 + childhood covariates				Model 3: model 2 + concurrent covariates			
	FT work		PT work		FT work		PT work		FT work		PT work	
	RRR	95% CI	RRR	95% CI	RRR	95% CI	RRR	95% CI	RRR	95% CI	RRR	95% CI
*Work–family pattern*												
Work, early family	Ref		Ref		Ref		Ref		Ref		Ref	
Work, marriage, non-parent	0.65	0.35, 1.19	0.54	0.18, 1.59	0.65	0.35, 1.21	0.51	0.17, 1.49	0.54	0.29, 1.02	0.45	0.15, 1.32
Work, no family	**0.37**	**0.21, 0.66**	0.55	0.26, 1.18	**0.36**	**0.20, 0.65**	0.51	0.23, 1.13	**0.39**	**0.20, 0.74**	0.57	0.25, 1.28
Work, later family	0.82	0.56, 1.20	1.05	0.61, 1.81	0.88	0.59, 1.31	0.96	0.55, 1.68	0.95	0.62, 1.44	1.02	0.58, 1.79
*Childhood covariates*												
Father’s social class												
I/II (highest)					**1.88**	**1.15, 3.06**	2.05	0.98, 4.26	**1.79**	**1.07, 3.00**	1.89	0.90, 3.97
IIINM					1.01	0.61, 1.65	2.25	1.07, 4.74	0.92	0.53, 1.60	2.09	0.98, 4.44
IIIM					Ref		Ref		Ref		Ref	
IV/V (lowest)					**1.72**	**1.10, 2.69**	**2.90**	**1.50, 5.58**	**1.68**	**1.04, 2.71**	**2.76**	**1.43, 5.35**
Educational attainment												
No qualification					Ref		Ref		Ref		Ref	
O-level					0.83	0.51, 1.35	1.03	0.50, 2.12	0.76	0.44, 1.29	0.95	0.46, 1.97
A-level					0.65	0.41, 1.02	1.14	0.60, 2.19	**0.54**	**0.33, 0.89**	0.93	0.47, 1.84
Degree level					**0.47**	**0.26, 0.85**	1.38	0.60, 3.14	**0.38**	**0.20, 0.71**	1.05	0.45, 2.46
Poor adolescent mental health												
No					Ref		Ref		Ref		Ref	
Yes					0.92	0.55, 1.53	0.54	0.23, 1.27	0.81	0.46, 1.41	0.54	0.23, 1.28
Child illness requiring hospitalisation												
No					Ref		Ref		Ref		Ref	
Yes					0.94	0.59, 1.52	0.68	0.33, 1.41	1.04	0.62, 1.74	0.73	0.35, 1.55
*Concurrent covariates*												
Caregiving for ≥ 10 h/week at age 60–64												
No									Ref		Ref	
Yes									**0.45**	**0.23, 0.89**	**0.67**	**0.25, 1.77**
Housing tenure at age 60–64												
Own outright									Ref		Ref	
Mortgage									**2.56**	**1.62, 4.05**	1.10	0.55, 2.19
Rent/other									0.99	0.54, 1.83	0.54	0.19, 1.49
Limiting illness at age 60–64												
No									Ref		Ref	
Yes									**0.24**	**0.15, 0.40**	**0.42**	**0.22, 0.79**

*FT* full-time; *PT* part-time; RRR relative risk ratio; bold indicates *p* < 0.05

Men who were partnered but did not have children (‘work, marriage, non-parent’ pattern) were less likely to be in paid employment at age 68–69 (RRR = 0.25, 95% CI 0.09, 0.72) compared with those in the ‘work, early family’ pattern controlling for all childhood and adult covariates (Table 4) and less likely to be in full-time employment at age 60–64 (RRR = 0.54, 95% CI 0.29, 1.02; *p* = 0.06).

**Table 4:** Likelihood of being in employment at age 68–69; men (*n* = 875)

	Model 1: crude association		Model 2: model 1 + childhood covariates		Model 3: model 2 + concurrent covariates^a^	
	OR	95% CI	OR	95% CI	OR	95% CI
Work, early family	Ref		Ref		Ref	
Work, marriage, non-parent	0.46	0.19, 1.09	**0.26**	**0.09, 0.74**	**0.25**	**0.09, 0.72**
Work, no family	1.06	0.57, 1.98	0.87	0.45, 1.70	0.92	0.47, 1.81
Work, later family	0.80	0.53, 1.21	0.75	0.48, 1.16	0.74	0.47, 1.15

*OR* odds ratio; bold indicates *p* < 0.05

^a^Caring ≥ 10 h per week at age 68–69, housing tenure at age 68–69 and limiting illness at age 68–69

Women in the ‘work, no family’ pattern were significantly less likely to be in part-time work at age 60–64 (RRR = 0.30, 95% CI 0.12, 0.79; Table 5).

**Table 5:** Likelihood of being in full-time or part-time employment at age 60–64 (base outcome is not in employment); women (*n* = 1024)

	Model 1: crude association				Model 2: model 1 + childhood covariates				Model 3: model 2 + concurrent covariates			
	FT work		PT work		FT work		PT work		FT work		PT work	
	RRR	95% CI	RRR	95% CI	RRR	95% CI	RRR	95% CI	RRR	95% CI	RRR	95% CI
*Work–family pattern*												
Work, early family	Ref		Ref		Ref		Ref		Ref		Ref	
Work, marriage, non-parent	0.79	0.31, 2.00	0.63	0.27, 1.45	0.75	0.30, 1.90	0.66	0.28, 1.51	0.68	0.26, 1.82	0.63	0.27, 1.48
Work, no family	0.86	0.33, 2.24	**0.35**	**0.14, 0.88**	0.80	0.31, 2.08	**0.28**	**0.11, 0.72**	1.04	0.38, 2.80	**0.30**	**0.12, 0.79**
Work, later family	**4.95**	**1.68, 14.55**	1.47	0.40, 5.36	**5.08**	**1.73, 14.90**	1.45	0.37, 5.64	**5.36**	**1.84, 15.60**	1.50	0.38, 5.93
Later family, work break	1.21	0.52, 2.79	1.88	0.97, 3.64	1.13	0.46, 2.76	1.85	0.95, 3.59	1.16	0.48, 2.82	1.87	0.96, 3.66
Early family, work break	0.98	0.45, 2.13	0.86	0.42, 1.76	0.94	0.43, 2.07	0.87	0.43, 1.78	0.90	0.41, 2.00	0.87	0.42, 1.80
Early family, PT work	**0.40**	**0.18, 0.89**	1.19	0.67, 2.11	**0.40**	**0.18, 0.90**	1.34	0.76, 2.38	**0.40**	**0.18, 0.88**	1.38	0.78, 2.45
Early family, no paid work, early family	0.36	0.11, 1.20	0.55	0.23, 1.29	0.35	0.10, 1.21	0.60	0.25, 1.41	0.39	0.11, 1.38	0.63	0.27, 1.48
*Childhood covariates*												
Father’s social class												
I/II (professional/technical)					1.16	0.58, 2.31	**1.74**	**1.04, 2.91**	1.33	0.66, 2.66	**1.82**	**1.08, 3.07**
IIINM (skilled non-manual)					1.12	0.58, 2.16	1.04	0.59, 1.84	1.24	0.63, 2.44	1.08	0.60, 1.92
IIIM (skilled manual)					Ref		Ref		Ref		Ref	
IV/V (partly/unskilled manual)					0.73	0.38, 1.39	**1.86**	**1.15, 2.99**	0.73	0.38, 1.42	**1.88**	**1.16, 3.05**
Educational attainment												
No qualification					Ref		Ref		Ref		Ref	
O-level					1.71	0.97, 3.01	**2.00**	**1.26, 3.18**	1.69	0.93, 3.09	**2.02**	**1.26, 3.25**
A-level					0.76	0.35, 1.66	**2.13**	**1.24, 3.64**	0.68	0.30, 1.51	**2.10**	**1.21, 3.67**
Degree level					1.42	0.42, 4.79	**4.57**	**2.00, 10.44**	1.31	0.37, 4.61	**4.63**	**2.00, 10.71**
Poor adolescent mental health												
No					Ref		Ref		Ref		Ref	
Yes					1.23	0.65, 2.35	0.94	0.57, 1.53	1.19	0.63, 2.23	0.91	0.56, 1.49
Child illness requiring hospitalisation												
No					Ref		Ref		Ref		Ref	
Yes					0.79	0.38, 1.65	1.15	0.67, 1.96	0.83	0.38, 1.79	1.18	0.69, 2.02
*Concurrent covariates*												
Caregiving for ≥ 10 h/week at age 60–64												
No									Ref		Ref	
Yes									1.12	0.48, 2.61	1.29	0.69, 2.42
Housing tenure at age 60–64												
Own outright									Ref		Ref	
Mortgage									**3.01**	**1.72, 5.26**	**1.68**	**1.03, 2.75**
Rent/other									1.27	0.51, 3.16	1.20	0.63, 2.30
Limiting illness at age 60–64												
No									Ref		Ref	
Yes									**0.28**	**0.13, 0.60**	**0.61**	**0.38, 1.00**

*FT* full-time; *PT* part-time; RRR relative risk ratio; bold indicates *p* < 0.05

### Work and timing of family formation

Among men, there was no difference in later life employment participation by age at family formation. Women who had children later (‘work, later family’ pattern) were more likely to be in full-time work at age 60–64 (Table 5). This was not explained by childhood or concurrent covariates. In the fully adjusted model, they were 5.36 (95% CI 1.84, 15.60) times as likely to be in full-time work as women in the ‘work, early family’ pattern.

Women who formed their families early and did not return to work (the ‘early family, no paid work’ pattern) were less likely to be in full- or part-time work at age 60–64 (though this did not attain statistical significance). Women who formed their families early and returned to part-time work (the ‘early family, part-time work’) were less likely to be in full-time work at age 60–64. Women who formed their families early and returned to full-time work after a work break (the ‘early family, work break’ pattern) did not differ in the later life employment participation compared with the reference pattern.

### Covariates and family formation

Childless men (‘work, marriage, non-parent’ and ‘work, no family’ patterns) tended to come from more socioeconomically advantaged backgrounds and have higher educational attainment than the reference pattern, though men who did not partner or have children tended to have poorer childhood mental and physical health and lower rates of home ownership (Supplementary Tables C, D). Women who did not partner or have children also tended to have poorer childhood health and lower home ownership. Women in the ‘work, later family’ pattern tended to have more socioeconomically advantaged background and educational attainment, but more commonly had an outstanding mortgage on their home. Among women who formed their families early, those who returned to work on a part-time basis or did not return to work tended to have lower educational attainment, poor adolescent mental health and higher prevalence of limiting illness at age 60–64 compared with those in the ‘work, early family’ pattern.

## Discussion

Work and family patterns from age 16 to 51 derived by multichannel sequence analysis were associated with paid work participation at age 60 +. In this cohort of British baby-boomers, both men and women characterised by continuous full-time employment who neither partnered nor had children were less likely than their counterparts who formed a family early to be in paid work at age 60 +. This was seen for both full- and part-time work for men and primarily for part-time work for women. Among mothers, but not fathers, timing of family formation in combination with work through adulthood was related to later work participation. Adjustment for childhood illness and socioeconomic position, education, and later life caregiving, housing tenure, and limiting illness did not attenuate these associations.

We hypothesised that not having children would be associated with a lower likelihood of later life paid work and that this association would be stronger for men than women. Our results support this association but indicate similar effects for both genders, manifested in full-time employment among men and part-time employment (the predominant form of employment at this age) among women. Our findings align with previous studies set in the USA (Pienta 1999), Germany (Hank 2004), and Britain (Blekesaune et al. 2008) which have shown that women who have had children are more likely to work in later life, although this finding is not universal and may depend on national context (Hank and Korbmacher 2013). We extend those studies to include men as well as women who reached state pension age in the twenty-first century. The ‘work, no family’ and ‘work, early family’ patterns were both characterised by continuous full-time employment through ages 16–51. Further exploration indicated possible health disadvantage in childhood and at age 60 + among childless men and women. They tended to be more socioeconomically advantaged in childhood but not in adulthood (based on their housing tenure). Poor health is associated with lower likelihood of later life work participation (Clark et al. 2017; Disney et al. 2006; van Rijn et al. 2014), but having greater wealth is associated with lower motivation to continue working in later life (Banks and Smith 2006; Farnham and Sevak 2007). Adjustment for these covariates did not explain the differences between those who did not have children and the reference pattern although there may be residual confounding by poor emotional health or other vulnerabilities and fuller exploration of the explanatory pathways should be undertaken in future research. In particular, a more comprehensive assessment of wealth may explain the lower work participation of men without children, or it may be that lower wealth is not a strong determinant of extended working in this group. We are not aware that studies have considered the role of wealth in later life employment decisions by parenthood and marital status.

The other main finding of the current study is that timing of having children was related to women’s but not men’s later life employment. Women in the ‘work, later family’ pattern were considerably more likely to be in full-time work at age 60–64 than those in the ‘work, early family’ pattern. This is past state pension age for women but there was no evidence that later family formation was associated with employment status at age 68 for men either. Only 3.4% of women in this cohort formed their families late and worked in continuous full-time employment from age 16 to 51. The ‘later family, work break’ pattern also formed their families late but took a break from paid work during their thirties and early forties. Women in this work–family pattern were somewhat more likely to be in part-time work at age 60–64 compared with the reference. Put together, these findings show that women who formed their families later were more likely to be working in their sixties, though working hours in later life depended on changes to their working patterns during the childrearing phase. Women who have their children later may be more likely to work at older ages because they have a greater work orientation and more rewarding jobs (Hank 2004). An indication of this is reflected in their higher educational attainment compared with the ‘work, early family’ pattern (Supplementary Table D), though adjustment for education and other confounders did not explain the difference in work participation by timing of family formation. We did not have data on women’s priorities for work and family that would be needed to test this more fully. Economic motivations may also explain this finding. Those who have their children later may still be providing financial support, though in this case we would expect that men who had children later would also be more likely to be in paid employment in later life and this was not borne out in the data. Women who formed their families later may also have to started working later and therefore accrued less pension entitlement by age 60–64 for example because of longer time spent in education though as noted adjustment for educational attainment did not explain the differences seen here.

Among women who formed their families early, we found differences in later work participation according to their return to work following childrearing. Those who returned to full-time work after a work break did not differ in later life work participation from the reference pattern who worked continuously in full-time employment. By contrast, women who worked part-time after a work break were less likely to be working full-time in their sixties compared to the reference pattern, although did not differ in likelihood of later part-time employment. This suggests that women who do not resume their previous employment role and responsibilities before childrearing do not do so later in their career. Those who did not return to work after family formation were less likely to be in any employment in later life, though this did not attain statistical significance. This is expected, since women who have not returned to employment through mid-adulthood are unlikely to do so in their sixties and is in line with other studies showing continuity of work patterns across adulthood (Blekesaune et al. 2008; Pienta et al. 1994; Pienta 1999; Worts et al. 2016). However, there may be a subgroup of these women who would like to return to work after a homemaking break and initiatives to facilitate this could, in the long run, have implications for raising rates of extended working. The long-term implications of part-time working and work breaks for men’s later life work participation could not be investigated in this cohort due to small numbers of men who did not have continuous participation in full-time employment.

Increases in the state pension age and reductions in the benefits derived from many occupational pensions will require younger cohorts to work further into older age. Generalisability to subsequent generations is unclear given societal changes including changes in occupations, partnership formation and dissolution, age at childbearing, and housing affordability. Whether this results in differential associations between work–family patterns and later life work participation across birth cohorts remains to be seen. However, it is clear that the size of the ‘work, no family’ group, the least likely to be in paid work in their sixties in the current study, increased dramatically between the cohort of men and women born in Britain in 1946 compared to those born in 1970. At the same time, there was a substantial increase in the size of the ‘work, later family’ group of women, the most likely to be in paid work in their sixties (McMunn et al. 2015). Generalisability to other nations is also unclear as there is country-level variation in policies throughout the life course, and particularly around retirement, which influence retirement transitions (Worts et al. 2016). Nevertheless, other Western nations have seen the same societal shifts and current evidence for Europe does not point to substantial national differences in work–family life courses (Fasang 2012; Madero-Cabib and Fasang 2016).

Several other work and family variables were not considered here. Partnership dissolution has sometimes (Damman et al. 2011) been linked to greater likelihood of later work participation though not all studies find this in multiply-adjusted models (Blekesaune et al. 2008). Older age at entry into employment also predicts later work participation (Blekesaune et al. 2008), but there was little variation in this in the current data and so we did not differentiate work–family patterns on this. The quality of work or family relationships and work–family conflict were not considered. We were unable to consider the increasingly complex transitions from working life to permanent retirement (Ekerdt 2010). We derived work–family patterns to age 51 to ensure that retirement transitions were not included in the exposure measurement as far as possible. Family transitions after age 51 were not considered, though only 196 partnership transitions occurred between age 52 and 60–64 and there were negligible changes to parenthood status. Other limitations include attrition due to death and drop-out. Whilst we know that not being married, poor health and work disability are associated with non-response in this study, we have no evidence that those who are unemployed, retired or homemakers are less likely to respond (Stafford et al. 2013). The work–family patterns of those who were not followed up into their sixties may be different to those included in the current analysis and we caution against generalising these descriptive patterns beyond the current study, though the proportion of men and women in paid work at age 60–64 in our study was consistent with estimates from the Labour Force Survey (Department for Work and Pensions 2015). Finally, state pension age differed for men and women in this cohort and so our findings at age 60–64 (in fact, age 62–64 for most of the sample) cannot easily be compared for men and women. Results for men at age 68 showed broadly the same association with work–family patterns as those at age 60–64, however. Study strengths include use of prospective data on work and family spanning 35 years and adjustment for prospective measures of childhood illness and socioeconomic background and proximal determinants of later life employment in a general population sample.

In the context of increasing life expectancy and concerns in many developed nations about how to fund state pensions, we set out to identify factors related to later life participation in paid work. We identified two work–family patterns that are associated with lower work participation in later life and of interest for further investigation. The first comprises men and women with no family. It does not appear that illness in childhood or later life explains their lower work participation. Future research might investigate other factors, including financial incentives, which might operate differently for this work–family pattern compared with other work–family combinations. The second comprises women who formed their families early and then did not return to paid work. This is in contrast to the group who returned to part-time work after early family formation and who remained in the work–force (in part-time jobs) in their sixties, and in contrast to the group who had a work break after early family formation but returned to full-time work. Job opportunities following family formation are important for the extending working lives agenda to consider. Mothers, and all parents more generally, may be retained in the work–force in later life if they can access jobs which allow them to combine family and work. This includes part-time jobs, examined here, and flexible working. The statutory right to request flexible working was not in place when this cohort formed their families though is now granted to all employees in the UK. Initiatives targeting parents who have taken a work break may be another approach to encouraging later life work participation rates. To some extent, these differences may be due to internal work versus family orientation of the women who did not return to work after forming their families. However, we note that rates of women returning to work after having children is context-dependent and shows considerable variation between periods and countries (Lyberaki et al. 2013).

## Electronic supplementary material

Below is the link to the electronic supplementary material.

Supplementary material 1 (DOCX 34 kb)
