# Supplementary material for: Work–family life course patterns and work participation in later life
Source: Eur J Ageing. 2018 Apr 2;16(1):83–94. doi: 10.1007/s10433-018-0470-7 (PMC6397104; doi:10.1007/s10433-018-0470-7)
Supplement: Supplementary file 1 — Supplementary material 1 (DOCX 34 kb) [file 10433_2018_470_MOESM1_ESM.docx]

Supplementary Table A. Duration in each work, partnership and parenthood status by work-family pattern: men

| **Mean years (SD)** | **Work, early family** | **Work, marriage, non-parent** | **Work, no family** | **Work, later family** |
| --- | --- | --- | --- | --- |
| Full-time employed | 31.7 (4.5) | 30.6 (5.3) | 25.5 (10.4) | 31.2 (4.2) |
| Part-time employed | 0.6 (2.1) | 0.6 (2.6) | 1.2 (3.0) | 0.6 (1.9) |
| Not employed | 2.6 (3.9) | 3.4 (4.4) | 7.9 (10.1) | 3.1 (3.5) |
| Married | 28.8 (3.5) | 24.7 (4.4) | 3.1 (5.5) | 24.9 (6.2) |
| Cohabiting | 0.4 (2.2) | 0.6 (2.7) | 1.9 (5.3) | 1.3 (5.7) |
| Not living with partner | 5.8 (2.6) | 9.7 (3.8) | 29.9 (7.0) | 8.8 (3.4) |
| No children under 17 years in household | 13.6 (3.0) | 32.5 (5.3) | 32.0 (6.4) | 13.4 (4.6) |
| Youngest child age 0-4 years in household | 10.3 (2.8) | 1.4 (2.9) | 1.9 (4.0) | 11.7 (4.0) |
| Youngest child aged 5-16 in household | 11.2 (1.1) | 1.1 (3.0) | 1.1 (3.0) | 10.0 (2.5) |

NB mean years in homemaking activities not shown for men

Supplementary Table B. Duration in each work, partnership and parenthood status by work-family pattern: women

| **Mean years (SD)** | **Work, early family** | **Work, marriage, non-parent** | **Work, no family** | **Work, later family** | **Later family, work break** | **Early family, work break** | **Early family, PT work** | **Early family, no paid work** |
| --- | --- | --- | --- | --- | --- | --- | --- | --- |
| Full-time employed | 25.8 (7.0) | 25.7 (8.8) | 25.0 (11.8) | 24.6 (8.0) | 12.3 (6.6) | 14.7 (4.9) | 5.9 (5.2) | 4.7 (4.9) |
| Part-time employed | 5.8 (6.2) | 5.7 (7.3) | 2.7 (5.3) | 3.0 (5.1) | 9.8 (6.2) | 6.8 (6.3) | 22.0 (6.8) | 7.7 (7.3) |
| Homemaking | 2.2 (3.2) | 2.1 (3.8) | 1.3 (3.4) | 5.3 (5.2) | 11.2 (5.7) | 12.3 (6.0) | 6.2 (5.8) | 19.8 (7.5) |
| Not employed | 1.2 (3.2) | 1.5 (3.6) | 6.0 (11.2) | 2.1 (6.0) | 1.6 (3.7) | 1.2 (3.1) | 0.9 (2.4) | 2.8 (5.1) |
| Married | 29.9 (4.8) | 26.9 (5.7) | 3.4 (6.5) | 24.1 (7.5) | 26.0 (5.1) | 29.8 (4.1) | 29.9 (3.4) | 28.2 (9.3) |
| Cohabiting | 0.6 (2.8) | 0.5 (2.7) | 1.4 (5.3) | 1.9 (7.3) | 0.6 (3.9) | 0.5 (3.2) | 0.3 (2.1) | 2.3 (8.1) |
| Not living with partner | 4.6 (3.5) | 7.6 (5.0) | 30.2 (8.0) | 9.0 (4.6) | 8.4 (3.6) | 4.6 (2.4) | 4.8 (2.8) | 4.5 (5.1) |
| No children under 17 years in household | 13.5 (3.3) | 29.2 (8.6) | 31.6 (7.1) | 11.0 (5.5) | 13.2 (4.0) | 12.2 (3.1) | 13.3 (4.2) | 11.5 (4.6) |
| Youngest child age 0-4 years in household | 10.2 (2.9) | 2.4 (3.6) | 1.8 (3.7) | 12.1 (4.1) | 11.4 (3.5) | 11.6 (3.0) | 10.6 (3.2) | 12.4 (4.0) |
| Youngest child aged 5-16 in household | 11.2 (0.9) | 3.4 (5.1) | 1.6 (3.6) | 11.9 (2.8) | 10.3 (1.9) | 11.1 (0.9) | 11.0 (1.9) | 11.1 (2.1) |

Supplementary Table C. Childhood and adult characteristics by work-family pattern: men

|  | **Work, early family** | **Work, marriage,**  **non-parent** | **Work, no family** | **Work, later family** |
| --- | --- | --- | --- | --- |
|  | % | % | % | % |
| **Childhood covariates** |  |  |  |  |
| Father's social class |  |  |  |  |
| I/II (highest) | 20.2 | 27.8 | 33.5 | 28.3 |
| IIINM | 17.1 | 17.2 | 15.3 | 24.9 |
| IIIM | 32.4 | 29.1 | 25.0 | 28.3 |
| IV/V (lowest) | 30.2 | 25.9 | 26.3 | 18.5 |
| Educational attainment |  |  |  |  |
| No qualification | 37.7 | 31.7 | 35.6 | 22.7 |
| O-level | 22.8 | 18.6 | 16.1 | 21.8 |
| A-level | 26.8 | 31.0 | 27.1 | 32.7 |
| Degree level | 12.6 | 18.7 | 21.2 | 22.9 |
| Poor adolescent mental health |  |  |  |  |
| No | 85.5 | 82.5 | 76.3 | 85.7 |
| Yes | 14.5 | 17.6 | 23.7 | 14.3 |
| Child illness requiring hospitalisation |  |  |  |  |
| No | 84.5 | 88.5 | 78.7 | 87.1 |
| Yes | 15.6 | 11.5 | 21.3 | 12.9 |
| **Concurrent covariates** |  |  |  |  |
| Caregiving for ≥10hrs/week at age 60-64 |  |  |  |  |
| No | 92.3 | 91.2 | 91.1 | 93.4 |
| Yes | 7.7 | 8.8 | 8.9 | 6.6 |
| Caregiving for ≥10hrs/week at age 68-69 |  |  |  |  |
| No | 92.5 | 90.1 | 95.8 | 94.4 |
| Yes | 7.5 | 10.0 | 4.2 | 5.6 |
| Housing tenure at age 60-64 |  |  |  |  |
| Own outright | 67.7 | 68.6 | 62.0 | 67.8 |
| Mortgage | 24.7 | 21.7 | 18.8 | 25.1 |
| Rent/other | 7.6 | 9.7 | 19.3 | 7.2 |
| Housing tenure at age 68-69 |  |  |  |  |
| Own outright | 84.3 | 83.6 | 72.0 | 87.7 |
| Mortgage | 8.1 | 8.5 | 6.7 | 4.2 |
| Rent/other | 7.6 | 7.9 | 21.3 | 8.1 |
| Limiting illness at age 60-64 |  |  |  |  |
| No | 78.0 | 83.9 | 69.7 | 75.3 |
| Yes | 22.0 | 16.1 | 30.3 | 24.7 |
| Limiting illness at age 68-69 |  |  |  |  |
| No | 57.5 | 62.5 | 56.9 | 61.4 |
| Yes | 42.5 | 37.5 | 43.1 | 38.6 |

Supplementary Table D. Childhood and adult characteristics by work-family pattern: women

|  | **Work, early family** | **Work, marriage, non-parent** | **Work, no family** | **Work, later family** | **Later family, work break** | **Early family, work break** | **Early family,**  **PT work** | **Early family,**  **no paid work** |
| --- | --- | --- | --- | --- | --- | --- | --- | --- |
|  | % | % | % | % | % | % | % | % |
| **Childhood covariates** |  |  |  |  |  |  |  |  |
| Father's social class |  |  |  |  |  |  |  |  |
| I/II (highest) | 18.9 | 21.0 | 40.5 | 40.3 | 29.5 | 20.2 | 23.0 | 26.0 |
| IIINM | 27.5 | 15.4 | 26.5 | 21.6 | 21.1 | 22.3 | 16.5 | 17.8 |
| IIIM | 30.5 | 43.3 | 14.8 | 26.5 | 25.3 | 24.3 | 33.6 | 30.4 |
| IV/V (lowest) | 23.2 | 20.3 | 18.2 | 11.7 | 24.1 | 33.1 | 26.9 | 25.8 |
| Educational attainment |  |  |  |  |  |  |  |  |
| No qualification | 27.0 | 26.9 | 20.7 | 22.7 | 19.8 | 34.0 | 43.4 | 45.8 |
| O-level | 40.4 | 42.3 | 30.4 | 37.3 | 33.4 | 44.2 | 32.0 | 33.4 |
| A-level | 28.8 | 27.6 | 34.3 | 36.6 | 37.9 | 18.9 | 19.2 | 15.9 |
| Degree level | 3.7 | 3.2 | 14.6 | 3.4 | 8.9 | 2.9 | 5.4 | 4.9 |
| Poor adolescent mental health |  |  |  |  |  |  |  |  |
| No | 86.7 | 80.2 | 72.9 | 86.4 | 67.9 | 81.4 | 77.2 | 78.1 |
| Yes | 13.3 | 19.8 | 27.1 | 13.6 | 32.1 | 18.6 | 22.8 | 21.9 |
| Child illness requiring hospitalisation |  |  |  |  |  |  |  |  |
| No | 87.0 | 78.8 | 78.5 | 87.5 | 89.8 | 88.9 | 85.8 | 81.7 |
| Yes | 13.0 | 21.2 | 21.5 | 12.5 | 10.2 | 11.1 | 14.2 | 18.3 |
| **Concurrent covariates** |  |  |  |  |  |  |  |  |
| Caregiving for ≥10hrs/week at age 60-64 |  |  |  |  |  |  |  |  |
| No | 90.9 | 81.7 | 86.0 | 86.3 | 88.1 | 91.5 | 92.1 | 82.5 |
| Yes | 9.2 | 18.4 | 14.0 | 13.7 | 11.9 | 8.5 | 7.9 | 17.5 |
| Housing tenure at age 60-64 |  |  |  |  |  |  |  |  |
| Own outright | 76.0 | 75.3 | 73.2 | 65.1 | 77.8 | 74.5 | 76.8 | 73.6 |
| Mortgage | 17.5 | 19.8 | 10.1 | 24.2 | 16.3 | 19.3 | 14.8 | 11.7 |
| Rent/other | 6.5 | 4.9 | 16.7 | 10.8 | 5.9 | 6.3 | 8.5 | 14.7 |
| Limiting illness at age 60-64 |  |  |  |  |  |  |  |  |
| No | 79.2 | 78.1 | 65.8 | 73.2 | 77.9 | 75.2 | 74.3 | 69.4 |
| Yes | 20.8 | 21.9 | 34.2 | 26.8 | 22.1 | 24.8 | 25.8 | 30.7 |
